# A Two-Phase Approach to the Use of Tafenoquine Was Associated with Cure of Babesiosis in Two Immunocompromised Patients with Relapsing Babesiosis

**DOI:** 10.3390/pathogens15090960

**Published:** 2026-09-09

**Authors:** Nonso Osakwe, Gary P. Wormser

**Affiliations:** 1 Department of Medicine, Division of Infectious Diseases, Northern Westchester Hospital-Northwell Health, Mount Kisco, NY 10549, USA; 2 Department of Medicine, Division of Infectious Diseases, New York Medical College, Valhalla, NY 10595, USA; gwormser@nymc.edu

**Keywords:** tafenoquine, immunocompromised, babesiosis, *Babesia microti*

## Abstract

Two immunocompromised patients with relapsing babesiosis were cured using a well-tolerated course of tafenoquine (171 and 212 days, respectively), with negative polymerase chain reactivity intentionally exceeding the 120-day lifespan of erythrocytes before discontinuing the tafenoquine. During the first 10 days of tafenoquine both patients also received an atovaquone-containing drug regimen. This was prescribed based on the demonstrated benefit of atovaquone on the efficacy of tafenoquine for completely eradicating *B. microti* in an animal study.

## 1. Key Points

1. Optimal treatment for *Babesia microti* infections in immunocompromised patients with relapsing infection has not been established.
2. We present two consecutive cases that may illustrate a successful and reliable approach for the use of tafenoquine to treat relapsing babesiosis in immunocompromised patients.
3. However, more studies are needed to confirm the efficacy and safety of this management approach.

## 2. Introduction

There is no established effective treatment regimen for highly immunocompromised patients with relapsing *Babesia microti* infection. We report on the use of a two-phase treatment regimen that was successfully used to treat two immunocompromised patients with relapsing babesiosis. The treatment regimen consisted of tafenoquine administered for at least 120 days after the patient became *B. microti* polymerase chain reaction negative, with the initial 10 days of tafenoquine therapy given in conjunction with a 10-day course of an anti-babesia combination drug regimen that included atovaquone.

## 3. Case #1

A 63-year-old Caucasian female was diagnosed with rheumatoid arthritis and Sjögren’s syndrome in June 2023. She was started on methotrexate, and then leflunomide. The patient had a progressive drop in hemoglobin: from 11.3 g/dL on 26 June 2023 to 8.3 g/dL on 7 August 2023, thought to be from an autoimmune hemolytic anemia (AIHA) associated with rheumatoid arthritis. Evaluation revealed a positive Coombs IgG consistent with AIHA.

The patient started on prednisone on 12 October 2023 but had continued hemolysis. Rituximab treatment was started on 30 November 2023 with completion of four weekly doses.

She presented on 29 December 2023 for shortness of breath and fever. A Babesia spp. blood smear revealed approximately 0.67–0.77% infected red blood cells, and the *Babesia microti* blood polymerase chain reaction testing (PCR) was positive. She was treated with azithromycin 500 mg daily and atovaquone 750 mg twice daily starting on 29 December 2023 for 6 weeks, with negative blood smears from 10 January 2024 through 9 February 2024.

The patient continued to have recurrent anemia, and on 22 March 2024 had recurrent parasitemia (0.2%), prompting reinitiation of azithromycin and atovaquone for 108 days. She was hospitalized from 17 April 2024 to 23 April 2024 for fever and neutropenia with a positive Babesia spp. blood smear. The Babesia spp. PCR remained positive until at least 2 July 2024. She continued to require RBC transfusions every 2 to 3 weeks.

Tafenoquine was started on 8 July 2024 after normal Glucose-6-Phosphate Dehydrogenase (G6PD) screening. The tafenoquine dosing was 200 mg daily for 3 days followed by 200 mg weekly. Azithromycin plus atovaquone was continued for 10 days with tafenoquine and then discontinued. The first negative Babesia spp. PCR was documented on 18 July 2024.

Following her last RBC transfusion on 27 June 2024, the hemoglobin level increased from 8.1 g/dL on 5 July 2024 to 10.8 g/dL on 8 August 2024. Tafenoquine was continued through 25 December 2024 for a total of 171 days. She tolerated tafenoquine well and required no further RBC transfusions, with hemoglobin stabilizing above 12 g/dL.

**Figure 1:**
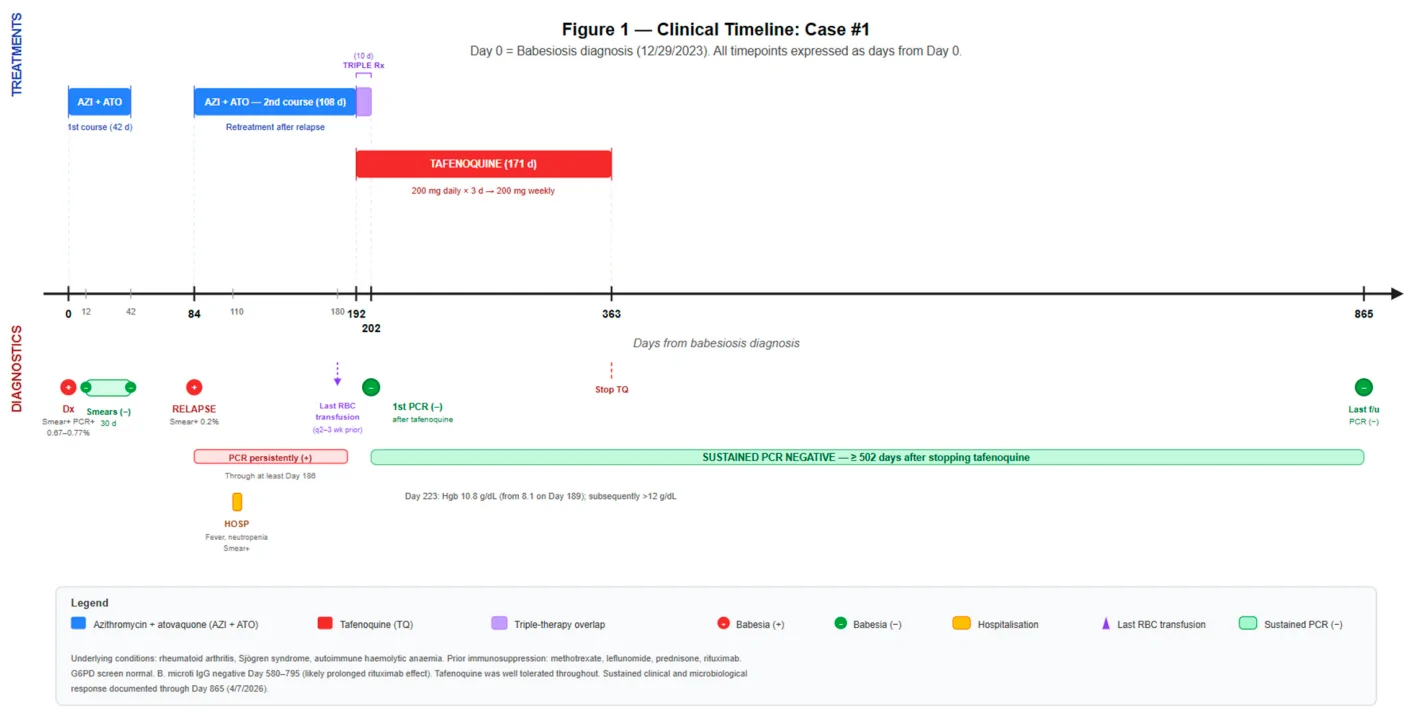
Clinical Timeline: Case#1. Day 0 = Babesiosis diagnosis date (29 December 2023). All timepoints expressed as days from Day 0.

She has experienced sustained symptomatic improvement through 7 April 2026, with persistent negative PCR including the last test which was performed on 7 April 2026. Thus, since discontinuing the tafenoquine, there have been at least 502 days with a documented negative Babesia spp. blood PCR. Babesia spp. antibody testing showed varying results over time but no titer of at least 512, as would be expected based on a large study of 245 babesiosis patients diagnosed in New York State using a polyvalent indirect immunofluorescence assay [1]. From 31 July 2025 to 3 March 2026 the *B. microti* IgG antibody testing was completely negative, consistent with the prolonged immunosuppressive effects of rituximab even after discontinuing this drug in December 2023. (Please see Table 1. Timeline for Case #1 and Figure 1).

## 4. Case #2

**Figure 2:**
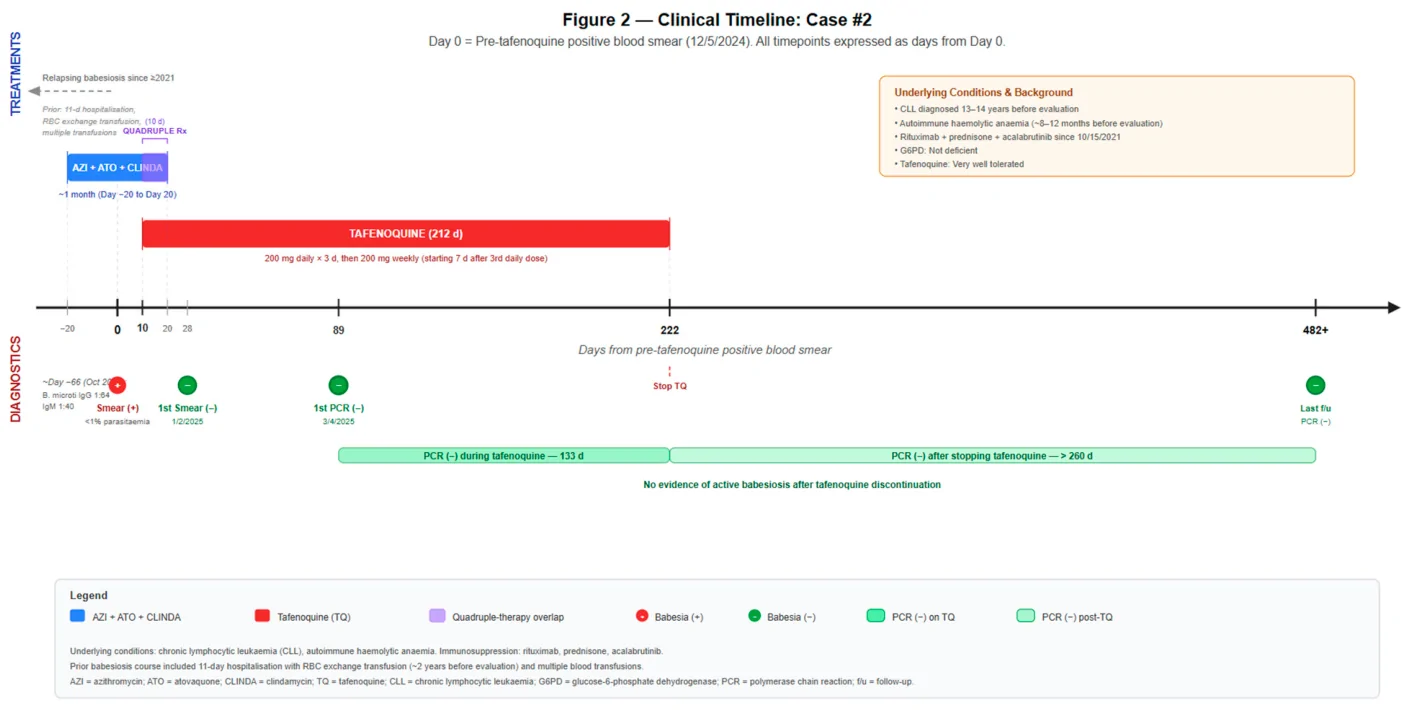
Clinical timeline: Case #2. Day 0 = Pre-tafenoquine positive blood smear (5 December 2024). All timepoints expressed as days from Day 0.

A 65-year-old immunocompromised Caucasian male was evaluated on 6 December 2024 because of relapsing babesiosis dating back to at least 2021. He had also been diagnosed with chronic lymphocytic leukemia (CLL) 13–14 years earlier and AIHA approximately 8–12 months prior to this evaluation. He had been receiving rituximab, prednisone and acalabrutinib since 15 October 2021. Approximately 2 years before this evaluation he had been hospitalized for babesiosis for 11 days and received a red blood cell exchange transfusion. He had received multiple other blood transfusions prior to the 6 December 2024 visit. He was being re-treated with azithromycin, atovaquone and clindamycin over the prior 1 month. In October 2024 the *B. microti* IgG antibody level was 1:64 and the IgM 1:40. On 5 December 2024, the Babesia spp. blood smear was positive at <1%. He was not G6PD deficient, and therefore on 15 December 2024 tafenoquine was started. He took 200 mg daily for three days and then 200 mg weekly beginning 7 days after the third 200 mg daily dose. The three-drug anti-babesiosis regimen that included atovaquone plus azithromycin plus clindamycin was discontinued after taking 10 days of this regimen concomitantly with the tafenoquine. The first *B. microti* PCR that tested negative was from a blood specimen obtained on 4 March 2025. The first negative blood smear was obtained on 2 January 2025. Tafenoquine was very well tolerated and was discontinued after the patient had received a total course of 212 days, during which PCR was negative for 133 days. Since stopping the tafenoquine, the patient has had no evidence of active babesiosis and has tested negative by PCR for over 260 additional days. (Please see Table 2. Timeline for Case #2 and Figure 2).

## 5. Discussion

A case series on the use of tafenoquine for relapsing babesiosis was published in 2024 [2]. Although the authors claim it consisted of five cases, one had been previously reported [3], and another one was deemed unevaluable. In only one case tafenoquine was used alone, and relapse occurred. In addition, the weekly maintenance dose of tafenoquine was 300 mg for three of the cases [2], despite the lack of data on tafenoquine drug safety over longer time intervals, in contrast to the 200 mg dose for which a 1-year study evaluating and establishing safety had been published [4]. Follow-up PCR testing after the discontinuation of tafenoquine was quite limited and ranged from only 1 to 8 months (two cases for 1 month, one case for 2 months and one for 8 months). Three of these four cases had received rituximab; however, Babesia spp. antibody testing was not reported [5].

The authors concluded that successful use of tafenoquine for relapsing babesiosis depends on concurrent treatment with other anti-*B. microti* drug therapies [2]. Despite the absence of any controlled treatment trials, the authors further stated that tafenoquine monotherapy is not well suited for treatment of persistent babesiosis in immunocompromised patients.

Clearly our results differ from these somewhat arbitrary predictions [2]. *B. microti* clearance is influenced by erythrocyte biology. Babesia spp. parasites reside intracellularly, and complete eradication may not be able to occur until all the infected erythrocytes, whose lifespan is approximately 120 days, are naturally removed from circulation [6]. If this does not happen, a biologic reservoir might persist even when parasitemia appears microscopically undetectable.

## 6. Conclusions

In conclusion, although tafenoquine has been highly effective in suppressing *B. microti* even in highly immunodeficient animal models [7,8,9,10,11], it per se does not appear to eradicate residual parasites present in erythrocytes [7], which is why we extended the tafenoquine treatment for our patients to achieve documented PCR negativity for more than 120 days, i.e., to reach and exceed the estimated 120-day lifespan of human erythrocytes. A 120-day erythrocyte lifespan should not be considered definitive proof of Babesia spp. eradication, but rather a biological reference point when interpreting sustained PCR negativity.

Furthermore, in an experimental model of *B. microti* infection with an atovaquone sensitive strain, Vydyam et al. [10] found that tafenoquine combined with atovaquone achieved radical cure and even conferred sterile immunity, suggesting that combination regimens may simultaneously target active parasites and residual intraerythrocytic reservoirs [10]. This observation led us to try a tafenoquine-plus-atovaquone combined approach at the initiation of tafenoquine therapy for our patients. Whether it was the prolonged tafenoquine treatment in which there was documented negative blood PCR for over 120 days while on tafenoquine or the initial use of atovaquone in combination with tafenoquine that unequivocally resulted in the eradication of Babesiosis in our two patients is unclear. Until the role of atovaquone per se is better understood, we recommend using both treatment approaches concomitantly if possible, as was done for these two patients during the first 10 days of tafenoquine therapy.

Of interest, although Babesiosis is characterized by non-immune hemolysis that resolves with antiparasitic drug therapy, studies have shown that *B. microti* infections may also lead to a secondary AIHA [12,13,14,15]. These studies raise the question of whether Babesiosis may have caused what was interpreted to have been an AIHA in both of our patients.

## 7. Challenge Question

An immunocompromised patient with relapsing *Babesia microti* infection remained PCR-positive despite multiple prior anti-babesia drug regimens. Tafenoquine was started and combined with an atovaquone containing regimen for the first 10 days of therapy. Tafenoquine was then continued for several months, with treatment intentionally extended until PCR negativity exceeded the estimated 120-day lifespan of erythrocytes. Which of the following describes the rationale for this management strategy?

A. A short course of atovaquone was used only to reduce tafenoquine toxicity, while the prolonged course of tafenoquine compensated for poor gastrointestinal absorption.
B. Combination therapy early in the treatment course may improve tafenoquine-induced *Babesia microti* parasite clearance and extending the tafenoquine therapy beyond the 120 day erythrocyte lifespan may potentially eradicate persistent intraerythrocytic infection in immunocompromised patients.
C. Tafenoquine therapy was continued long-term primarily to prevent emergence of drug resistance during therapy.
D. Prolonged anti-babesiosis drug therapy is required because *Babesia microti* forms latent hepatic stages that reactivate after treatment.
E. Therapy was extended mainly to suppress extracellular parasites until immune recovery occurred.

The correct answer is B. Relapsing babesiosis in immunocompromised patients may result from a persistent intraerythrocytic infection and incomplete parasite clearance. Experimental data suggest that atovaquone may enhance tafenoquine efficacy when given early in therapy. Continuing tafenoquine until the duration of PCR negativity exceeds the approximately 120-day-long erythrocyte lifespan may help to eliminate any residual infected erythrocytes and thereby reduce the risk of relapse of babesiosis.

## Figures and Tables

**Table 1:** Timeline for Case #1.

Event	Findings
Underlying conditions	Rheumatoid arthritis; Sjögren’s syndrome; autoimmune hemolytic anemia
Immunosuppressive therapies before babesiosis	Methotrexate, leflunomide, prednisone, rituximab
Evidence for autoimmune hemolytic anemia	Progressive anemia; Coombs IgG positive
Initial babesiosis diagnosis date	29 December 2023
Initial parasitemia	~0.67–0.77% infected RBCs on smear
Microbiologic confirmation	*B. microti* PCR positive
Initial anti-babesial drug therapy	Azithromycin 500 mg daily + atovaquone 750 mg twice daily for 6 weeks
Initial response	Blood smears negative from 10 January 2024 through 9 February 2024
Relapse date	22 March 2024
Relapse parasitemia	0.2%
Retreatment before tafenoquine	Azithromycin + atovaquone for 108 days
Complicated course	Hospitalization (17 April 2024–23 April 2024) for fever, neutropenia, positive Babesia blood smear
Duration of PCR positivity	Persistently positive until at least 2 July 2024
Transfusion requirement	RBC transfusions every 2–3 weeks before tafenoquine
Tafenoquine eligibility	Normal G6PD screen
Tafenoquine regimen	Start date 8 July 2024; 200 mg daily for 3 days, then 200 mg weekly
Combination period with standard therapy	Tafenoquine overlapped with azithromycin/atovaquone for 10 days
First negative PCR after tafenoquine	18 July 2024
Total tafenoquine duration	171 days (8 July 2024–25 December 2024)
Tafenoquine tolerability	Well tolerated
Hematologic recovery	Hgb 8.1 g/dL on 5 July 2024 to 10.8 g/dL on 8 August 2024; subsequently stabilized > 12 g/dL
Transfusion outcome	No further RBC transfusions after 27 June 2024
Long-term outcome	Sustained symptomatic improvement through 7 April 2026
Durability of microbiologic response	At least 502 days of documented negative blood Babesia PCR after stopping tafenoquine
Serologic observation	*B. microti* IgG negative from 31 July 2025 to 3 March 2026, likely reflecting prolonged rituximab effect

**Table 2:** Timeline for Case #2.

Event	Findings
Underlying conditions	Chronic lymphocytic leukemia diagnosed 13–14 years earlier and autoimmune hemolytic anemia diagnosed approximately 8–12 months before the initial evaluation by an author of this manuscript
Immunosuppressive therapy	Rituximab, prednisone, and acalabrutinib since 15 October 2021
Duration of babesiosis history	Relapsing babesiosis dating back to at least 2021
Prior severe babesiosis event	Approximately 2 years before evaluation, hospitalized for 11 days and treated with red blood cell exchange transfusion
Transfusion history	Multiple other blood transfusions before the 6 December 2024 visit
Retreatment before tafenoquine	Azithromycin, atovaquone, and clindamycin for approximately 1 month before tafenoquine initiation
Serologic findings	In October 2024, *B. microti* IgG was 1:64 and IgM was 1:40
Pre-tafenoquine parasitemia	Babesia blood smear on 5 December 2024 positive at <1%
G6PD status	Not G6PD deficient
Tafenoquine start date	15 December 2024
Tafenoquine regimen	200 mg daily for 3 days, then 200 mg weekly beginning 7 days after the third 200 mg daily dose
Concomitant therapy overlap	Atovaquone plus azithromycin plus clindamycin continued for 10 days with tafenoquine, then discontinued
First negative blood smear	2 January 2025
First negative *B. microti* PCR	Blood specimen obtained on 4 March 2025
Tafenoquine tolerability	Very well tolerated
Total tafenoquine duration	212 days
PCR-negative period during tafenoquine	PCR remained negative for 133 days while receiving tafenoquine
Post-tafenoquine outcome	No evidence of active babesiosis after tafenoquine discontinuation
Durability of response after discontinuation	Babesia PCR remained negative for more than 260 additional days after stopping tafenoquine

## Data Availability

Data is available on request.
